# Photocatalytic Activity of Fibrous Ti/Ce Oxides Obtained by Hydrothermal Impregnation of Short Flax Fibers

**DOI:** 10.3390/molecules26113399

**Published:** 2021-06-03

**Authors:** Mikhail F. Butman, Nataliya E. Kochkina, Nikolay L. Ovchinnikov, Karl W. Krämer

**Affiliations:** 1 Department of Ceramics Technology and Nanomaterials, Ivanovo State University of Chemistry and Technology, Sheremetevsky Avenue 7, 153000 Ivanovo, Russia; butman@isuct.ru (M.F.B.); ovchinnikovnl_1972@mail.ru (N.L.O.); 2 G.A. Krestov Institute of Solution Chemistry of Russian Academy of Sciences, Akademicheskaya St. 1, 153045 Ivanovo, Russia; 3 Department of Chemistry and Biochemistry, University of Bern, Freiestrasse 3, 3012 Bern, Switzerland; karl.kraemer@dcb.unibe.ch

**Keywords:** Ti/Ce oxides, Ti/Ce polyhydroxy complexes, photocatalytic activity

## Abstract

Fibrous Ti/Ce oxide photocatalysts were prepared for the first time by a biomimetic solution process using short flax fibers (flax straw processing waste) as a biotemplate. Titanium polyhydroxy complex solutions with 3% and 5% cerium were used as precursors. Flax fibers were impregnated in an autoclave under hydrothermal conditions. Ti/Ce oxides were obtained from the biotemplate by annealing at 600 °C. The photocatalytic activity of the Ti/Ce oxides was studied by the adsorption and decomposition of the dye rhodamine B under UV irradiation. The photocatalytic decomposition of the dye was 50% and 75% faster for Ti/Ce oxides with 3% and 5% Ce, respectively, than for the analogous undoped fibrous TiO_2_. The morphologies, textures, and structures of the photocatalysts were studied by scanning electron microscopy, low temperature N_2_ adsorption/desorption, UV-Vis spectroscopy, and X-ray and XPS analytical methods. It was shown that the introduction of Ce into the precursor solution increased the surface irregularity of the Ti/Ce oxide crystallites compared to pure TiO_2_. This effect scaled with the Ce concentration. Ce improved the UV light absorption of the material. The Ti/Ce oxides contained Ce^4+^/Ce^3+^ pairs that played an important role in redox processes and intensified the photocatalytic activity.

## 1. Introduction

Titanium oxide TiO_2_, or titania, is an intensely studied, highly effective photoactive material and photocatalyst [1,2]. Various synthetic methods have been proposed to improve the photoactivity of TiO_2_, based on a modified morphology of the material or doping of TiO_2_ with different elements [3,4,5]. For example, the biomimetic method uses natural templates, in particular cellulose, to form photocatalytical particles with a hierarchical structure. The particles have a large surface area, with structural elements ranging from nano- to micrometer size, and contain micro-, meso-, and macropores [6]. Such structures promote the diffusion and adsorption of reagent molecules, as well as light absorption, which improves the TiO_2_ photocatalytic activity [7,8].

The effectiveness of TiO_2_ photocatalysts strongly depends on the probability of the recombination of electron–hole pairs generated on the particle surface by the absorption of photons. Doping TiO_2_ with metal ions lowers the probability of electron–hole recombination and improves interphase charge transfer, thus increasing the photocatalytic efficiency [3,9]. Researchers have proposed a number of photocatalytic TiO_2_ systems mixed with ZnO [10], Cu_2_O [11], ZrO_2_ [12], SnO_2_ [13], WO_3_ [14], and CeO_2_ [15,16,17,18,19,20,21,22,23,24,25,26].

CeO_2_ is an especially attractive dopant for TiO_2_ due to its stability at high temperatures, strong UV absorption, optical transparency in the visible spectral range, chemical stability, and redox reactivity [27]. The most common method for Ti/Ce oxides is a sol–gel synthesis [15,16,17,18]. Besides, some authors apply chemical coprecipitation–peptization methods [19,20] and hydrothermal syntheses [21,22]. Ti/Ce oxides in the form of 3D structures with a specific surface area were prepared by oil-in-water (o/w) [23] and water-in-oil (w/o) suspension techniques [24]. However, there are only a few reported biotemplated syntheses of Ti/Ce oxides. For example, Xiao et al. [25] prepared cerium-doped TiO_2_ mesoporous nanofibers from Ti (SO_4_)_2_ and Ce(SO_4_)_2_ precursor solutions and collagen fiber templates. The use of collagen fibers in biotemplated synthesis is quite reasonable because they represent a valuable material for various biomedical applications. Wang et al. [26] synthesized CeO_2_-TiO_2_ samples by impregnating filter paper with Ce(NO_3_)_3_·6H_2_O and TiCl_4_ precursor solutions, followed by annealing of the biotemplate. The stability of filter paper in the acidic precursor solutions is extremely low, which makes this preparation method of biomorphic ceramic material rather difficult.

We have earlier reported a method for the preparation of TiO_2_ using short flax fibers as a template. As is known, the amount of waste in flax straw processing can reach 75%, as only long flax fibers are used for making linen fabrics, and short fibers are not suitable for these purposes [28]. Flax fibers have a multimeric structure with pores and capillaries and possess higher acid resistance than other cellulose materials; they are most suitable for the biomimetic synthesis of ceramic fibers based on strongly acidic precursor solutions. We used a solution of titanium polyhydroxy complexes as precursor because it is better adsorbed by cellulose biotemplates than the other precursor systems. An intensive impregnation of flax fibers with a solution of titanium polyhydroxy complexes under hydrothermal conditions facilitates the nucleation of photoactive precursor particles and the thermal destruction of the biotemplate when it is annealed during the subsequent TiO_2_ synthesis. The obtained samples of fibrous TiO_2_ have a better photocatalytic activity than commercially available photocatalysts and their known analogues.

This work is a continuation of our series of studies on obtaining highly effective photocatalysts by the biotemplate method. For the first time, we investigated titanium and cerium hydroxy complexes as precursor solutions to impregnate short flax fibers. The aim of the present study was to establish how a small addition of a cerium salt in the precursor solution affects the photocatalytic activity of the synthesized systems, as well as their morphology, phase composition, and textural characteristics.

## 2. Results and Discussion

The catalysts were synthesized from mixed solutions of Ti–Ce hydroxy complexes, see Section 3.1 for details. The solutions were hydrothermally impregnated on short flax fibers. The calcination of the fibers resulted in biotemplated Ti/Ce oxides.

**Figure 1:**
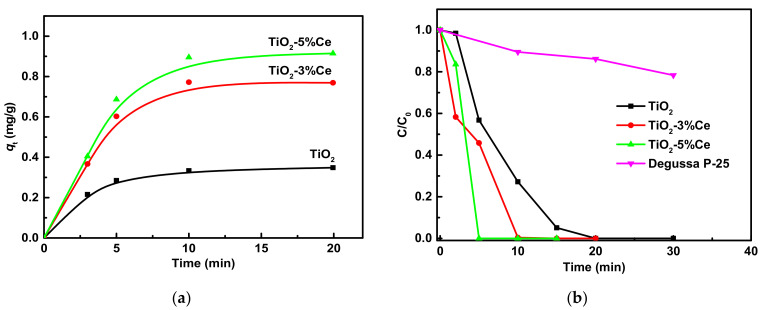
Kinetic curves of rhodamine B adsorption by fibrous photocatalysts at 25 °C (**a**) and photocatalytic degradation of the dye under UV irradiation (**b**).

Bearing in mind the aim of the study, we first tested the photocatalytic properties of the synthesized TiO_2_, TiO_2_–3% Ce, and TiO_2_–5% Ce samples. Figure 1a,b show the results of the rhodamine B adsorption on the catalysts and the photocatalytical decomposition of the dye by UV irradiation, respectively. 

Photocatalysis is the additive result of two processes: the adsorption and photocatalytic degradation of organic molecules. Figure 1 shows the time-dependent adsorption of the dye rhodamine B by the synthesized samples. The amount of rhodamine B adsorbed by the TiO_2_–3% Ce and TiO_2_–5% Ce samples was much higher than for pure TiO_2_. Saturation was achieved after about 10 min and the maximum adsorbed values increased from 0.34 to 0.77 and 0.91 mg/g for TiO_2_, TiO_2_–3% Ce, and TiO_2_–5% Ce, respectively. The dye decomposition under UV irradiation took 50% and 75% less time in the presence of TiO_2_–3% Ce and TiO_2_–5% Ce catalysts compared to pure TiO_2_, see Figure 1b. These results agreed well with the data obtained in earlier work on photocatalysts synthesized by the sol–gel method [18]—raising the cerium concentration to 5% in mixed Ti–Ce oxides increased their activity in organic pollutant decomposition. Impressively, the materials synthesized in this work showed a higher photocatalytic activity than the commercial photocatalyst P-25 from Degussa.

In the next step, we studied the effect of the cerium addition to the titanium hydroxy complexes on the structure of the biomorphic photocatalysts to rationalize the higher photocatalytic activity of the Ti–Ce oxides compared to pure TiO_2_.

**Figure 2:**
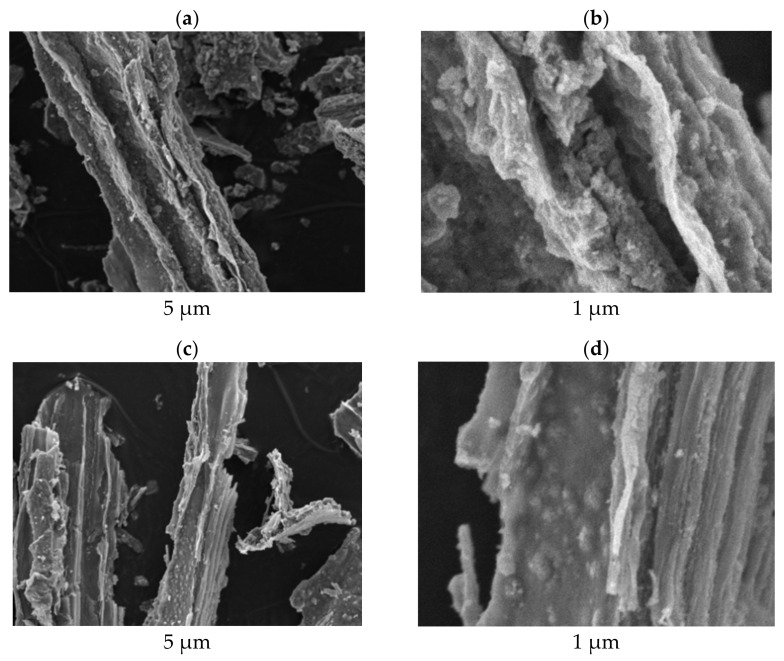
SEM images of fibrous TiO_2_–3% Ce (**a**,**b**) and TiO_2_–5% Ce (**c**,**d**).

The morphologies of the biotemplated TiO_2_–3% Ce and TiO_2_–5% Ce samples were studied by SEM, see Figure 2. The SEM images of flax fiber and TiO_2_ samples were published previously in [28]. The particles of all the photocatalysts had a similar fibrous structure replicating the texture of the flax template. The samples consisted of agglomerated elongated crystallites with pores of different sizes between them.

**Figure 3:**
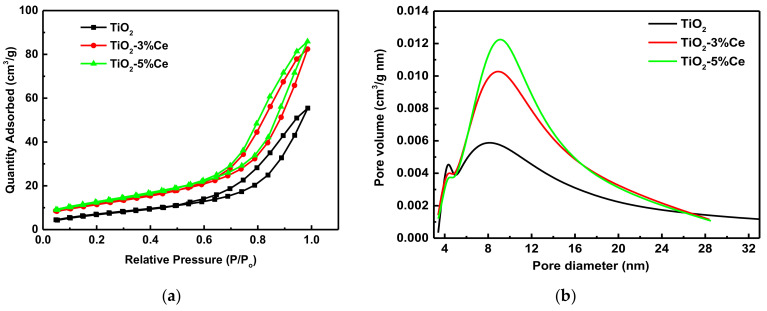
Nitrogen adsorption/desorption isotherms (**a**) and pore size distribution (**b**) of the fibrous pure TiO_2_ and TiO_2_–xCe samples.

The porosity of the photocatalysts was studied by low temperature N_2_ adsorption/desorption measurements, which are presented in Figure 3 and Table 1.

The nitrogen adsorption isotherms presented in Figure 3 can be classified as type IV with an H3-type hysteresis loop (according to the IUPAC classification), which is typical of mesoporous materials [29]. The use of a mixed Ti–Ce solution of hydroxy complexes in the synthesis of photocatalysts increases their specific surface area and porosity. The values of all the parameters characterizing the texture of the studied materials become higher with increasing Ce concentrations.

To explain these observations, we calculated the fractal dimensions (*D*_F_) using the nitrogen adsorption results according to the FHH (Frenkel–Halsey–Hill) model [30]. The FHH isotherm equation in the logarithmic form looks as follows:

ln*V* = const + (*D*_F_ − 3) ln(−ln*P*/*P*_0_)

where *V* is the volume of the adsorbate gas under equilibrium pressure, *P*.

**Figure 4:**
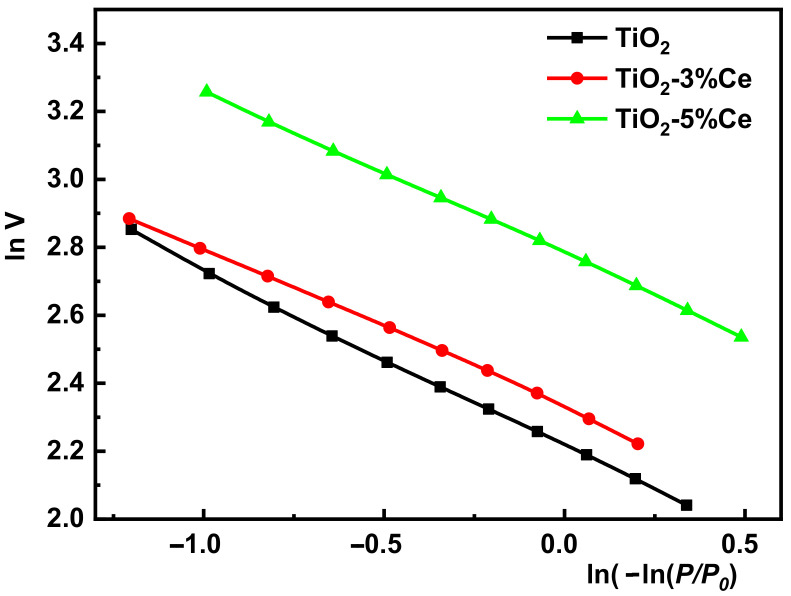
Dependence of ln*V* on ln(−ln*P*/*P*_0_) for the fibrous TiO_2_ and TiO_2_–xCe samples.

It is evident that the slope of the ln*V* − ln(−ln*P*/*P*_0_) dependence is equal to *D*_F_ − 3. The dependence is shown in Figure 4; the calculated *D*_F_ values are given in Table 1.

As is known, the higher the *D*_F_ value (ranging from 2 to 3), the more irregular is the surface. The *D*_F_ values were higher in the cerium-containing samples of the photocatalysts obtained in this work. As a result, the crystallites making up their inner structure had a more irregular surface than those in pure TiO_2_.

One possible reason for the higher porosity and more developed inner surface of the Ti–Ce oxide samples is the formation of coordination complexes by Ce and Ti hydroxy complexes of various sizes. Such effects observed during the hydrothermal synthesis of large Al-Ce hydroxy complexes were earlier reported in [31]. As in the present work, the *D*_F_ values of the pillared montmorillonite crystallite ensemble became higher when small cerium additives were introduced into the aluminum hydroxy complexes, acting as intercalants.

Thus, the texture of the photocatalysts explains the increasing adsorption of rhodamine B along the series TiO_2_ < TiO_2_–3% Ce < TiO_2_–5% Ce.

**Figure 5:**
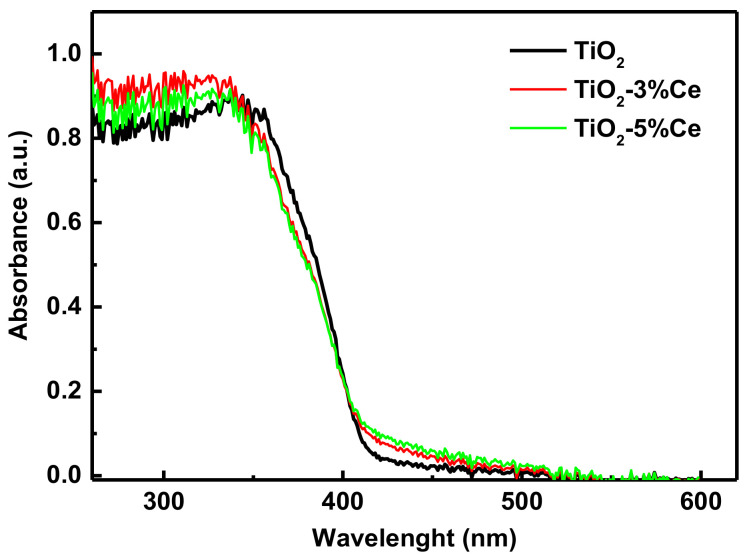
UV–Vis absorption of the fibrous TiO_2_ and TiO_2_–xCe samples.

The UV–Vis absorption spectra of the samples are shown in Figure 5. The TiO_2_–3% Ce and TiO_2_–5% Ce samples showed a stronger (up to 10%) blue/UV light absorption than pure TiO_2_. It is evident that a higher UV light absorption by the Ce-doped systems intensifies the formation of electron–hole pairs during the photocatalysis [32] and partly explains why the catalytic properties of the Ti–Ce oxide samples were better than those of pure TiO_2_. Ce^4+^ compounds are often yellow, which agrees with the higher absorption from 400–470 nm. Ce^3+^ compounds showed a strong 4f-5d absorption in the UV. Both facts indicate a stronger absorption of the Ti–Ce oxides compared to Ti oxide.

We found the band gap of the samples under study by the Tauc method [33] to be 3.16 ± 0.1, 3.15 ± 0.1, and 3.13 ± 0.1 eV for TiO_2_, TiO_2_–3% Ce and TiO_2_–5% Ce, respectively. So, the doping technique applied did not yield any statistically significant changes in the band gap.

**Figure 6:**
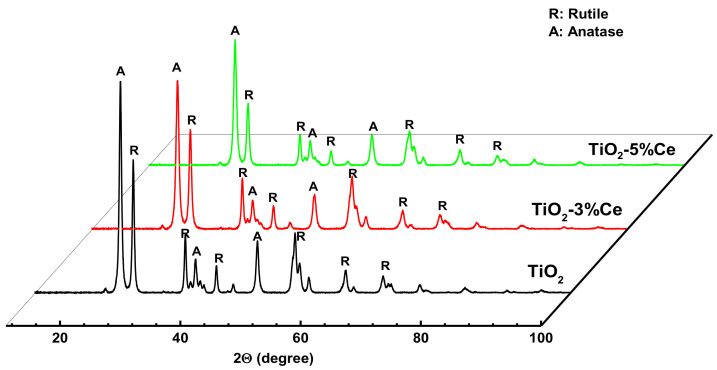
XRD patterns of the fibrous TiO_2_ and TiO_2_–x% Ce samples.

The X-ray diffraction (XRD) patterns of the samples are shown in Figure 6. 

All samples showed peaks of rutile (R) and anatase (A) TiO_2_ phases, as indicated in the diffraction patterns. No diffraction peaks of cerium oxides were observed. Similar results for mixed Ti–Ce oxides with up to 10 wt.% Ce were obtained by the authors of [18]. They supposed that the formation of nanosized cerium oxide crystallites well dispersed among the TiO_2_ crystallites is, evidently, a reasonable explanation as to why no characteristic cerium oxide peaks were observed in the diffraction patterns of the mixed materials. It is noteworthy that mixed Ti/Ce oxides incorporating both Ce^4+^ and Ce^3+^ [34] may also form in these systems. The DIFFRAC.SUITE data base provided by the Bruker Company (Karlsruhe, Germany), which we used to evaluate the X-ray patterns, confirmed such probability. However, Ti/Ce oxide peaks, which cannot demonstrate high enough intensity to obtain information due to the low concentration of cerium, overlapped with the TiO_2_ ones, and thus it was impossible to obtain reliable information.

The average crystallite size of the TiO_2_ phases, as calculated by the Scherrer’s equation, and the relative amount of A and R phases are summarized in Table 2.

The data demonstrated that the introduction of Ce into titanium hydroxy complexes somewhat increased the rutile content in the TiO_2_–Ce samples. Also, it reduced the size of the crystallites for both TiO_2_ phases. A higher rutile content in TiO_2_–Ce samples agrees with the earlier results in [35]. The researchers attributed this fact to the lower activation energy of the anatase–rutile phase transition in the presence of a dopant. 

**Figure 7:**
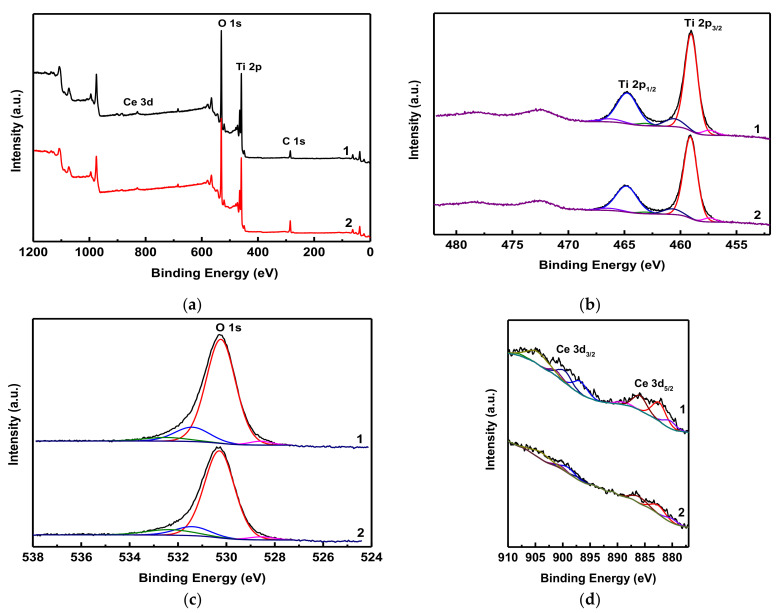
XPS survey spectra (**a**), Ti 2p XPS spectra (**b**), O 1s spectra (**c**), and Ce 3d region (**d**) of the TiO_2_–5% Ce sample before (1) and after (2) the photocatalysis.

To evaluate the chemical state of Ti, O, and Ce on the photocatalyst surface in the mixed Ti–Ce oxide system and to understand the mechanism of dye decomposition under the action of UV radiation in the presence of a Ti–Ce photocatalyst, we carried out an XPS analysis of the TiO_2_–5% Ce sample that demonstrated the best photocatalytic activity. The system was studied before and after the photocatalysis. Figure 7a presents the XPS survey spectra of the TiO_2_–5% Ce sample. Both spectra clearly showed binding energy peaks characteristic of Ti 2p, O 1s, Ce 3d, and C 1s. It should be noted that the presence of the C element in the sample could be the result of incomplete biotemplate burning during the heat treatment. 

The XPS spectra of the Ti 2p core level are shown in Figure 7b. The spectra had two main peaks at 459.0 and 464.7 eV corresponding to the spin–orbit components of Ti 2p_3/2_ and Ti 2p_1/2_, respectively. The positions of these peaks indicate that the TiO_2_–5% Ce sample contains Ti in the form of Ti^4+^ both before and after the photocatalysis. Figure 7c illustrates the O 1s spectra of the TiO_2_–5% Ce samples. The peak at 530.2 eV in these spectra is characteristic of the lattice oxygen in the structures of TiO_2_ and CeO_2_. Figure 7d shows the core level Ce 3d spectra with their fitted deconvolution. The spectra included four Ce 3d_5/2_ peaks: two of them—at 882 and 889 eV—corresponded to Ce^4+^ and the other two—at 880 and 885 eV—to Ce^3+^. The Ce 3d_3/2_ peaks at 899 and 907 eV indicated Ce^4+^.

Then. we calculated the Ce^4+^/Ce^3+^ ratio by comparing the total areas under the peaks corresponding to Ce^4+^ and Ce^3+^. The Ce^4+^/Ce^3+^ ratio of the initial TiO_2_–5% Ce sample was found to be 6.1/3.9. After this same sample was subjected to photocatalysis, the Ce^4+^/Ce^3+^ ratio became 5.3/4.7. It means that a certain amount of Ce^4+^ had been reduced to Ce^3+^ as a result of the exposure to ultraviolet irradiation and the photocatalytic process itself. 

The obtained XPS results allowed us to make certain conclusions about the photocatalysis mechanism in the presence of the synthesized Ti–Ce oxide photocatalysts.

The authors of [23,36] have shown that Ce^4+^/Ce^3+^ pairs increase the efficiency of the photocatalytic process as follows:

Ultraviolet effects:

TiO_2_ + hν → TiO_2_ (e^−^ + h^+^) (1)

Redox process on the surface:

Ce^4+^ + e^−^ → Ce^3+^ (2)

Formation of active particles/radicals:

Ce^3+^ + O_2_ →·O_2_^−^ + Ce^4+^ (3)

O_2_^−^ + H^+^ → ·HO_2_ (4)

HO_2_ +·HO_2_ → H_2_O_2_ + O_2_ (5)

e^−^ + H_2_O_2_ → HO· + OH^−^ (6)

H_2_O_2_ + O_2_^−^ → HO· + OH^−^ + O_2_ (7)

Photocatalytic destruction of the dye:

Rhodamine B + radical → products of Rhodamine B destruction(8)

Consequently, it can be assumed that cerium oxide crystallites dispersed among the TiO_2_ in the synthesized Ti–Ce oxides promote the photocatalytic process due to: (i) a stronger UV light absorption and, as a result, the formation of more electron–hole pairs and (ii) Ce^3+^/Ce^4+^ redox processes (2) and (3).

It is quite probable that the rate of exciton generation (1) and subsequent reduction (2) was higher than that of the redox reaction (3), which was limited by diffusion and adsorption. As a result, we observed a higher Ce^3+^ content on the surface of the Ti–Ce photocatalyst after its participation in the photocatalysis.

## 3. Materials and Methods

### 3.1. Preparation of Photocatalyst Samples by the Biotemplate Method

The precursor solutions with titanium and cerium polyhydroxy complexes were prepared in the following way. First, we made a solution of titanium hydroxy complexes by hydrolyzing titanium chloride TiCl_4_ (Sigma-AldrichRus, Moscow, Russia) at room temperature by the technique described in [28]. For that purpose, we added TiCl_4_ dropwise to a 6M HCl solution until the Ti^4+^ concentration reached 4.92 M. Then, this solution was slowly diluted with distilled water under constant stirring until the Ti^4+^ concentration was 0.56 M. This solution was aged for 3 h at 20 °C leading to the formation of titanium polyhydroxy complexes. At the next stage, we added a solution of Ce(NO_3_)_3_∙6H_2_O of an appropriate concentration to the titanium hydroxy complex solution under constant stirring at 20 °C to obtain 3 and 5 wt.% of CeO_2_ in the final catalysts.

Short flax fibers were used as a biotemplate in this work [28]. The biotemplate was impregnated with the precursor solutions in an autoclave for 5 h at 115 °C and a pressure of 170 kPa. Then, the autoclave was cooled passively to room temperature. The samples were separated from the precursor solution in a centrifuge at a circumferential velocity of 1500 rpm and dried at 95 °C until a constant weight was reached. The fibrous photocatalysts were prepared by calcination of the impregnated biotemplates in air in an electric furnace at 600 °C for 30 min. The calcination temperature was in accordance with our previous study that revealed the best photocatalytic activity for fibrous TiO_2_ samples was prepared at 600 °C [28]. The Ti/Ce oxides denoted as TiO_2_–3% Ce and TiO_2_–5% Ce, where 3 and 5 wt.% were the Ce(NO_3_)_3_∙6H_2_O concentrations in the precursor solutions.

### 3.2. Study of the Structure and Properties of the Photocatalyst Samples

The surface morphologies of the TiO_2_ samples were studied on a VEGA3 scanning electron microscope (TESCAN, Brno, Czech Republic). The textural characteristics and the average pore diameter of the samples were determined by low-temperature nitrogen adsorption–desorption on a NOVAtouch LX specific surface and porosity analyzer (Quantachrome, Boynton Beach, FL, USA); the samples were degassed for 3 h at 120 °C and a residual pressure of 1.3 Pa before the measurements.

The UV–Vis spectra of the samples were recorded on an AvaSpec-ULS2048x64-EVO spectrometer (Avantes, Apeldoorn, The Netherlands).

X-ray diffraction patterns were measured with Cu Kα radiation on a D2 PHASER diffractometer (Bruker, Karlsruhe, Germany). The average crystallite size (*L*) of the catalysts anatase and rutile phases were determined by the Scherrer method [37]:

$$L=\frac{k\lambda }{\beta \cos\theta }$$

where *k* is the dimensionless particle shape coefficient (0.94), *λ* is the X-ray wavelength (*λ* = 0.15425 nm), *β* is the full width at half maximum of the diffraction peak (in 2*θ* units), and *θ* is the diffraction angle. 

The elemental composition and chemical state of the surface of the obtained samples were evaluated by X-ray photoelectron spectroscopy (XPS) on an ES2403 electron spectrometer (Institute for Analytic Instrumentation of RAS, St Petersburg, Russia) with an XR-50 X-ray source (SPECS, Berlin, Germany), a Mg Kα anode (hν = 1253.6 eV), and a PHOIBOS 100-MCD5 energy analyzer (SPECS, Berlin, Germany). The survey spectra were recorded with an energy step of 0.5 eV and an analyzer pass energy of 40 eV, while the high-resolution spectra were recorded with an energy step of 0.05 eV and an analyzer pass energy of 7 eV. The spectra were deconvoluted using the CasaXPS software (Casa Software Ltd, Teignmouth, UK).

The textural and X-ray measurements were repeated thrice for each photocatalyst sample; the resulting mean values and uncertainties are given in Table 1 and Table 2.

### 3.3. Evaluation of the Photocatalytic Activity of the Samples

The photocatalytic activity of the obtained fibrous TiO_2_ samples was evaluated by studying the decomposition of the dye rhodamine B [28] in an aqueous solution under UV radiation. The UV source was a 250 W high-pressure mercury lamp (Philips, Royal Philips Electronics, Amsterdam, The Netherlands) with a radiation peak at 365 nm. The lamp in a water-cooled quartz jacket was placed into the center of an 800 mL reaction vessel. A magnetic stirrer at the bottom of the reactor ensured an effective mixing of the reaction components. The reaction solution was purged with air at a constant rate to maintain a steady concentration of the oxygen. In each of the experiments, we added 0.3 g (0.6 g/L) of the photocatalyst powder sample to 500 mL of the dye solution with a concentration of 12 mg/L. The reaction mixture was stirred at 25 °C. For analysis, 3 mL samples of the suspension were taken out of the mixture at certain time intervals; the photocatalyst was separated from the dye solution in a centrifuge at 8000 rpm for 15 min. The dye concentrations in the solutions, before and after the processing in the photocatalytic reactor, were determined photometrically on a Hitachi U-2001 spectrometer (Hitachi, Tokyo, Japan) by measuring the optical density at the wavelength λ_max_ = 554 nm, which corresponded to the RhB absorption maximum. A preliminary irradiation of the dye solution for 1 h without photocatalyst revealed no changes in the optical density.

The adsorption of rhodamine B by the photocatalyst was studied under the same conditions as for the photocatalytic activity but without UV irradiation. The amount of the adsorbed dye (*q_t_,* mg/g) on the sample over time *t* was calculated by the equation:

$$q_{t}=\frac{(C_{0}-C_{t})V}{m}$$

where $C_{0}$ and $C_{t}$ (mg/L) are the initial dye concentration and its concentration at the time *t* (min), *V* is the dye solution volume (L), *m* is the weight of the air-dry adsorbent sample (g).

All the adsorption and photocatalytic experiments were repeated twice.

## 4. Conclusions

We presented the synthesis of fibrous Ti/Ce oxides and investigated their photocatalytic activity. Short flax fibers (flax processing waste) were used as template and impregnated by hydrothermal treatment with a solution of Ti hydroxy complexes containing a small additions (3% and 5%) of cerium. Subsequent calcination in air at 600 °C produced Ti/Ce oxides with improved adsorption and photocatalytic properties compared to pure TiO_2_. The adsorption and photocatalytic decomposition of Rhodamine B was investigated; the doping by cerium significantly enhanced the dye decomposition rate. All the fibrous oxides replicated the texture of the flax template. At the same time, the Ti/Ce oxides were characterized by a more developed surface than TiO_2_, which may have resulted from the formation of coordinated hydroxy complexes by Ce and Ti of various sizes in precursor solutions. The cerium inclusion in the structure of the photocatalysts increased their UV light absorption to some extent. The XPS results showed the presence of redox-active Ce^4+^/Ce^3+^ pairs on the surface of the Ti/Ce oxide fibers. These pairs actively participated in the formation of radicals, which destroyed the dye molecules. 

## Figures and Tables

**Table 1:** Porosity data for the fibrous TiO_2_ and TiO_2_–xCe samples.

Sample	*S*_BET_ (Mesopores) (m^2^/g)	*V*_BJH_ (cm^3^/g)	*V*_Total_ (cm^3^/g)	*D*_p_ (nm)	*D*_F_
TiO_2_	27.1 ± 1.4	0.088 ± 0.001	0.086 ± 0.001	7.7 ± 0.9	2.483 ± 0.006
TiO_2_–3% Ce	42.9 ± 2.1	0.122 ± 0.001	0.128 ± 0.001	9.3 ± 1.1	2.515 ± 0.008
TiO_2_–5% Ce	46.0 ± 2.3	0.127 ± 0.001	0.133 ± 0.001	9.4 ± 1.1	2.534 ± 0.009

**Table 2:** Average crystallite size and phase composition of the fibrous TiO_2_ and TiO_2_–x% Ce samples.

Sample	Average Crystallite Size, nm		Phase Composition, %	
	A	R	A	R
TiO_2_	21.0 ± 1.6	26.4 ± 2.0	51.3 ± 1.0	48.7 ± 0.9
TiO_2_–3% Ce	17.5 ± 1.5	20.8 ± 1.6	47.3 ± 0.9	52.7 ± 1.0
TiO_2_–5% Ce	17.4 ± 1.5	22.8 ± 1.8	46.6 ± 0.9	53.4 ± 1.1

## Data Availability

Available from the authors.

## Sample Availability

Samples of the compounds are available from the authors.
